# An exploratory study of resting-state functional connectivity of amygdala subregions in posttraumatic stress disorder following trauma in adulthood

**DOI:** 10.1038/s41598-022-13395-8

**Published:** 2022-06-10

**Authors:** Leticia Leite, Nathalia Bianchini Esper, José Roberto M. Lopes Junior, Diogo Rizzato Lara, Augusto Buchweitz

**Affiliations:** 1 School of Medicine, Pontifical Catholic University of Rio Grande do Sul (PUCRS), Porto Alegre, 90619-900 Brazil; 2 School of Psychology and Health, Federal University of Health Sciences of Porto Alegre (UFCSPA), Porto Alegre, 90050-170 Brazil; 3 Cingulo Mental Health App, Porto Alegre, 90430-001 Brazil; 4 Brain Institute (BraIns), Pontifical Catholic University of Rio Grande do Sul (PUCRS), Porto Alegre, 90610-000 Brazil; 5 Department of Psychology, University of Connecticut, Stamford, 06269-1020 United States of America

**Keywords:** Post-traumatic stress disorder, Amygdala

## Abstract

We carried out an exploratory study aimed at identifying differences in resting-state functional connectivity for the amygdala and its subregions, right and left basolateral, centromedial and superficial nuclei, in patients with Posttraumatic Stress Disorder (PTSD), relative to controls. The study included 10 participants with PTSD following trauma in adulthood (9 females), and 10 controls (9 females). The results suggest PTSD was associated with a decreased (negative) functional connectivity between the superficial amygdala and posterior brain regions relative to controls. The differences were observed between right superficial amygdala and right fusiform gyrus, and between left superficial amygdala and left lingual and left middle occipital gyri. The results suggest that among PTSD patients, the worse the PTSD symptoms, the lower the connectivity. The results corroborate the fMRI literature that shows PTSD is associated with weaker amygdala functional connectivity with areas of the brain involved in sensory and perceptual processes. The results also suggest that though the patients traumatic experience occured in adulthood, the presence of early traumatic experiences were associated with negative connectivity between the centromedial amygdala and sensory and perceptual regions. We argue that the understanding of the mechanisms of PTSD symptoms, its behaviors and the effects on quality of life of patients may benefit from the investigation of brain function that underpins sensory and perceptual symptoms associated with the disorder.

## Introduction

Posttraumatic stress disorder (PTSD) is a major public health problem that affects veterans and prisoners of war, victims of psychological and physical abuse, victims of criminal violence, witnesses of such abuse and violence^1,2^. People with PTSD experience intrusive thoughts, mental images, nightmares and flashbacks. The re-experiencing of the trauma can lead to self-destructive and aggressive behaviors, irritability, trouble concentrating and it can have a general, negative effect on the quality of life. PTSD also leads to avoidance of stimuli that knowingly trigger re-experiencing the trauma^3,4^^.^ If the disorder is chronic, it can lead to increased risk of suicide and substance abuse^5^. PTSD is thus associated with symptoms and behaviors that compromise mental health. Understanding the interaction between the neural bases and PTSD symptoms and behaviors remains a challenge for a desired bridge between cognitive neuroscience and evidence-based mental health practices.

The goal of the present study was to explore functional brain connectivity alterations in the amygdala, and its subregions, and their association with posttraumatic stress disorder (PTSD) following trauma in adulthood. We carried out a cross-sectional, exploratory study of participants with PTSD and controls using resting-state fMRI (rs-fMRI); the PTSD group was made of adults whose experience that triggered PTSD occurred in adulthood.

Brain imaging studies have found PTSD to be associated with alterations in brain function, which include aberrant functional connectivity patterns in a neural substrate that includes anterior and posterior hippocampus and the amygdala^6–8^ ; PTSD has also been linked to hypoactivation of the medial prefrontal cortex, posterior insula and superior temporal gyrus, and to hyperactivation of the anterior insula and cerebellum^7,9^. There have also been consistent findings of alterations in brain function in the parahippocampal gyrus, anterior cingulate cortex, insula, and middle frontal gyrus^7,10,11^. Meta-analyses of fMRI studies of PTSD indicate the presence of (I) hypoactivation of the medial prefrontal cortex (mPFC), including the rostral and dorsal ACC^12^, (II) hyperactivation of the dorsal ACC and (III) hypoactivation of the frontal pre-ventromedial (vmPFC)^13^, and (IV) hypoactivation of the dorsal medial prefrontal cortex and hyperactivation of vmPFC^8^^.^ The degree to which regions of the brain are involved and whether there is more or less activation in brain regions in association with PTSD varies according to the type of trauma and the period of life (childhood, adulthood) in which the trauma occurred, and according to PTSD subtype^11^.

Functional brain imaging has only begun to unveil the complex host of brain regions associated with PTSD etiology, severity and symptoms. In the present study, we sought to focus on PTSD caused by trauma in adulthood, and to explore one of the key brain regions for processing emotions and stimuli and, also, for determining what one should do in response, i.e., the amygdala^14^. Amygdala response is associated with the severity of symptomatic states^15^, and it has been shown to predict PTSD symptom onset^16^.

The amygdala is central to affect-processing^17^. The amygdala and its sub regions are linked to integration of sensory and perceptual information, classical conditioning, social cognition, and reward processing^18^. The different ways in which the amygdala responds to affective stimuli mediate the complex neural processes that underpin affective behavior: Alterations in amygdala activation (e.g., hypoactivation) are associated with psychiatric problems that have difficulties processing fear and sadness, such as callous-unemotional traits and anxiety disorders^19–21^. Beyond its activation, how the amygdala communicates with other brain regions can be understood in terms of functional connectivity. In this sense, brain imaging studies have shown alterations in amygdala functional connectivity associated with anxiety disorders^22–24^, and with exposure to violence, early-life stress and institutionalization^25–27^.

The amygdala is a structurally and functionally heterogeneous nucleus complex of the brain^28,29^, which is subdivided into three main nuclei: basolateral amygdala (BLA), centromedial amygdala (CMA) and superficial amygdala (SFA). The basolateral amygdala (BLA) is associated with integrating sensory information and emotional valence^18^. The BLA is among the first subregions to process information in the amygdala^30^, it projects to a distributed network of cortical and subcortical structures such as the orbitofrontal cortex, the hippocampus and somatosensory areas^30,31^. The BLA is thus linked linked to associative learning^32^. The centromedial subregion, or CMA, in turn, is associated with relaying information gleaned from the BLA to the brainstem’s autonomic and motor centers; it is also associated with the brain mechanism that generates fear responses^33,34^. The superficial subregion, SFA, is adjacent to the olfactory cortex and it has been linked to how olfactory, social and affective information is processed, and to informing the ensuing behaviors^18,35–37^. SFA connectivity has been shown to change significantly in association with reduced PTSD symptomatology following neurofeedback^38^.

Resting-state functional magnetic resonance imaging (rs-fMRI) studies of the subregions in PTSD patients have consistently shown differences in connectivity patterns for the three regions^39–43^. Results show, on the one hand, stronger functional connectivity of the BLA with subgenual and dorsal ACC and dorsomedial prefrontal cortex in PTSD participants; on the other, they show weaker functional connectivity between BLA and the left inferior frontal gyrus in trauma- exposed controls. The decreased functional connectivity for the amygdala seems to be closely linked to PTSD symptom severity^11^. The amygdala is involved in a host of processes. Its complex connections with prefrontal cortical areas, for example, are involved with the level of cognitive control of emotions. In PTSD, these connections are central to explanations of the mechanisms that modulate symptom severity^40^.

Our goal was to explore functional connectivity patterns for all three subregions of the amygdala in association with PTSD symptoms caused by trauma in adulthood, compared to healthy controls. PTSD following trauma in adulthood is associated with alterations in frontolimbic brain function that may be comparable to a common set of alterations across mental health disorders, and are yet fully understood^25,44^. Our study focused on PTSD following adult trauma, but we also evaluated presence of trauma in early life, anxiety and depression. The heterogeneity of PTSD, its symptoms and behaviors are discussed, in turn, in relation to the associated alterations of functional connectivity.

## Methods

### Study design

We carried out a rs-fMRI case–control, exploratory cross-sectional study of PTSD following trauma in adulthood. Participants in the experimental group were matched with controls for age, sex, schooling and intellectual abilities (I.Q.).

### Participants

Twenty right-handed subjects participated in the study: 10 participants in the experimental group (9 females), who reported a traumatic experience in adult life and who presented provisional PTSD diagnosis (PTSD group); 10 participants in the control group (9 females), who had no symptoms of PTSD nor reported traumatic experiences (age range 18 to 60 years; mean age = 35.10 years; SD = 11.32 years). There were no statistically significant differences between the groups in terms of intelligence, schooling, or socioeconomic status (see Table 1). We had a prospective number of 38 study volunteers with PTSD. Yet, the number of participants who took part in the brain imaging study was limited for two main reasons: we excluded participants whose posttraumatic disorder was linked to an early childhood trauma (n = 23), and we excluded participants who had already sought psychotherapy treatment (n = 5). We established these criteria in order to have a more homogeneous experimental group. In retrospect, it limited our ability to find individuals that met the criteria within the timeframe of the study.

**Table 1:** PTSD and control evaluations (demographic, neuropsychological and trauma-related scores).

	PTSD (n = 10)		Control (n = 10)	
	Mean	SD ±	Mean	SD ±
Age (years)	35.1	11.2	35.1	11.5
Schooling (years)	15.7	2.6	15.9	2.4
SES	36.0	11.6	38.4	7.8
IQ	116.3	7.3	110.0	7.7
BAI	18.7*	7.7	5.5	3.8
PHQ-9	12.4*	4.3	4.1	4.3
CTQ	20.7**	11.3	9.6	5.0
PCL-5	43.6	10.9	–	–
fMRI head motion	0.053	0.03	0.054	0.001
Sex (female) n	9	–	9	–
Marital status (single) n	8	–	6	–

There were no statistically significant differences between the two groups for SES, IQ, and Schooling.

*SES* Socioeconomic status according to Associação Brasileira de Empresas de Pesquisa (www.abep.org): we report the final score, rather than the strata (letters A to D), *IQ* intelligence quotient, *BAI* beck anxiety inventory, *PHQ-9* Patient Health Questionnaire-9, *CTQ* Childhood Trauma Questionnaire—total score, *PCL-5* Posttraumatic checklist-5—total score, *SD* standard deviation.

*p-value < 0.001; **p-value < 0.02.

Individuals in the PTSD group were recruited by means of ads in academic and media platforms (social networks, websites, radio and television) over a period of one year. The trauma experienced by participants in the PTSD group were a result of armed robbery (n = 5), sexual abuse (n = 2), physical assault (n = 2) and domestic violence (n = 1). The traumatic episode occurred at least six months prior to the first data collection for PTSD participants. We did not obtain information on how far back the event had occurred, only that it occurred at least 6 months prior to the study. The control group included volunteers who had no current or previous diagnosis of psychiatric illness, and who did not report traumatic experiences in adult or early life (Structured clinical interview for DSM-5^45^ and Childhood Trauma Questionnaire^46^).

Exclusion criteria for both groups included: history of head injury, diagnosed neurological or degenerative disease, alcohol or drug abuse or dependence, safety contraindications for MRI scanning (metal implants, pacemakers and so on). For the PTSD group, we also excluded volunteers who reported psychotic symptoms. All PTSD group participants were making regular use of SSRIs antidepressants (selective serotonin reuptake inhibitors). We did not exclude PTSD participants who were using any class of psychiatry medication; however, we excluded PTSD participants who had changed their psychiatric medication regimen recently, i.e., up to 8 weeks prior to the evaluation. No PTSD group participants had had, or were undergoing psychotherapy. All PTSD group participants had had the traumatic experience that triggered the disorder in adulthood. Nonetheless, that the cause of PTSD was in adulthood does not necessarily mean that these participants had not had traumatic experiences in early childhood. Thus, we used the Childhood Trauma Questionnaire (CTQ)^46^ to assess early experiences. For the Control group, we excluded participants who presented diagnosis and/or current use of psychiatric medication, and who reported a potentially traumatic experience that fulfilled the A criterion for PTSD, according to the DSM-5^3^. The present study, and all of its instruments, methods and procedures were approved by the Research Ethics Committee of the Pontifical Catholic University of Rio Grande do Sul, which is in accordance with the Declaration of Helsinki (registration number CAEE 57,526,716.1.0000.5336). All participants gave their informed consent and signed an Informed Consent Form as approved by the Research Ethics Committee.

### Instruments for clinical evaluation

#### PTSD symptoms

We used the Posttraumatic Stress Disorder Checklist 5 (PCL-5)^47,48^ for the first evaluation of PTSD symptoms. The PCL-5 is a self-report instrument that assesses PTSD symptoms for the previous 30 days; it is based on DSM-5 criteria and gives provisional diagnosis, which was subsequently confirmed using Clinician-Administered PTSD Scale for DSM-5 (CAPS-5)^49^. CAPS was applied by a trained, experienced mental health professional to confirm PTSD diagnosis. The PCL-5 score for symptom severity ranges from 0 to 80. It is the sum of the response to 20 items in the checklist. The score for each item represents the participants rating on a five-point scale for severity of symptoms, which ranges from zero (not at all) to four (extreme). The cutoff for inclusion in the PTSD group was a score greater than or equal to 33 points. As stated previously, we subsequently confirmed PTSD by administering the CAPS. The choice of PCL-5 for screening was in line with using only self-reported instruments for the first evaluative steps in the study. All participants who had a score greater than or equal to 33 in PCL-5 later had their PTSD diagnoses confirmed by CAPS-5; however, 23 participants were excluded if their trauma had not been in adult life. Individuals with PTSD diagnosis were paired with healthy controls for sex, age, schooling (in total years) and IQ (see Table 1 for demographic and neuropsychological data).

#### Childhood trauma

We evaluated the history of child maltreatment using the Child Trauma Questionnaire (CTQ)^46^. The CTQ is a self-reported questionnaire that assesses five types of childhood trauma (emotional abuse, physical abuse, sexual abuse, emotional neglect, and physical neglect). The frequency of each type of trauma event is rated on a five-point scale that ranges from “never” to “always” for each of the 28 items, which are then scored from zero to four points, each.

#### Anxiety and depression symptoms

We used the Beck Anxiety Inventory (BAI)^50^ to evaluate anxiety symptoms. It is a questionnaire with 21 statements each of which describes a common anxiety symptom. Respondents have four alternatives; they are instructed to select the alternative that best describes the intensity they have experienced each symptom, over the previous week including the day of the evaluation. The statements are evaluated by the participant on a scale of zero to three, in which “0 = Not at all” and “3 = Severely—it bothered me a lot.” The final score ranges from 0 to 63. We used the Patient Health Questionnaire-9 (PHQ-9)^51^ to screen for symptoms of depression. The questionnaire includes nine questions for nine symptoms of depression. Responses are based on a four-point scale about the frequency in which symptoms occurred over the preceding 14 days. The frequency ranges from “not at all (0)” to “nearly every day (3)”. The final score ranges from 0 to 27.

#### Socioeconomic and Intelligence evaluations

We evaluated IQ using the Wechsler Abbreviated Scale of Intelligence (WASI)^52^. Socioeconomic status (SES) was scored based on a standardized questionnaire for SES classification in Brazil^53^, which provides a score based on schooling and possession of consumer goods. These scores are translated to an A-toD letter stratification that qualifies the SE strata, from A, the highest, to D, the lowest.

#### MRI and rs-fMRI acquisition

MR images were acquired using a 3.0 T GE Healthcare Signa HDxt scanner. Structural scans were acquired using the following parameters: T1 weighted, TE/TR = 6.16/2.18 ms, isotropic 1 mm3 voxels. Resting state fMRI images were acquired using the following parameters: T2* EPI BOLD: 29 interleaved axial slices, 3.6 mm slice thickness, 240 mm × 240 mm FOV and matrix size of 64 × 64, TE = 30 ms, TR = 2000 ms, flip angle of 90° for a total 210 volumes (7 min) (previous studies used similar protocols^42,68,100^ ). Resting State fMRI scans were acquired while participants were instructed to rest with their eyes open and fixating on a white “ + ”sign centrally projected against a black background on an LCD screen.

### Statistical analyses

#### fMRI analyses

We used AFNI's^54^ afni_proc.py to perform single subject image processing and group analysis. The preprocessing steps were carried out in the following order: removal of the first 3 TRs, despiking, slice-time correction, motion correction, band-pass filter (0.01–0.1 Hz), spatial normalization using the MNI152 template using nonlinear warping (T1 image as reference), and non-linear spatial normalization to 3.5 × 3.5 × 0.39 mm3. Images were subsequently blurred using a 6 mm-FWHM Gaussian kernel. Next, multiple regression was carried out on the functional data in which the average cerebrospinal fluid signal, the six motion parameters and their derivatives were used as nuisance regressors. Data points with motion > 0.3 mm were censored. The average head motion for all participants was 0.0281 mm (SD = 0.0337). The data points from the multiple regression were used in the connectivity analysis.

The criteria for exclusion was that TR’s with motion outliers > 0.3 mm were censored from the data. The criterion for participant exclusion from the study due to head motion was excessive motion in 20% or more of the TRs. There were no participants with excessive motion in 20% or more of the TRs (i.e. no participants were excluded due to excessive head motion). The average head motion for each group was PTSD M = 0.053 (SD = 0.03), Control M = 0.054 (SD = 0.001). There was no statistically significant difference in head motion between the PTSD and control groups (p = 0.917). Moreover, to ensure motion artifacts did not have an effect on the correlation among clinical scores and brain function, we calculated the correlation among participants' average head motion during the fMRI scan and their score for all scores. There were no significant correlations among the average movement in the scanner and CTQ (r = 0.3407; p = 0.1415), PCL-5 (r = 0.4850; p = 0.15), BAI (r = 0.4132; p = 0.23) and PHQ-9 (r = 0.5387; p = 0.10).

#### Resting-state fMRI analysis: amygdala seeds

Amygdala seeds were defined using the Juelich histological atlas implemented in FSL. The atlas defines basolateral (BLA), centromedial (CMA), and superficial (SFA) subdivisions based on stereotaxic and probabilistic maps of cytoarchitectonic boundaries^32,55^. All seeds included voxels with at least a 50% probability of belonging to their subdivision. A voxel with overlapping subdivision was assigned to the most likely region. We calculated the average of the time series for all voxels in each seed and generated the average of the time series^55^. As stated previously, the present study is exploratory and, hence, we investigated PTSD-associated amygdala subregions' seed connectivity differences over the whole brain.

#### Single-subject connectivity maps

The atlas provides six amygdala regions, three in each hemisphere. These regions were resampled to match the voxel size of the normalized functional data. We calculated the mean BOLD time-series within each amygdala region (*3dROIstats* AFNI command) and then used *3dTcorr1D* to generate a voxel-wise Pearson's correlation map for each region. We used Fisher's r to z transformation to prepare the maps for group analyses.

#### Group analyses

Group-level analyses for each connectivity map was carried out using a t-test and correlation analysis for all six amygdala subdivisions. We used the 3dClustSim program (estimate the blurring of the data by the autocorrelation function) to correct for multiple comparisons. We calculated the cluster threshold for a corrected p- score of ɑ < 0.05. The program estimated that a threshold of p < 0.005 and a minimum cluster size of 44 voxels (2102, 1 μl) were required for a correction for multiple comparisons for a corrected p- score of ɑ < 0.05. This estimation was applied to all group-level analyses.

We carried out correlations between the PCL-5, BAI, PHQ-9 and CTQ scores and individual connectivity map. The correlation was calculated using the *3dRegAna* function from the AFNI package^55^. There were no significant correlations among BAI and the connectivity maps. All group-level analyses were estimated with correction for multiple comparisons. We carried out three ANCOVAs for each of the six subregions: one ANCOVA for BAI, one for CTQ, and another for PHQ9. We also carried out one ANCOVA for each of the six subregions using a combination of all three scores (BAI, CTQ, and PHQ9) as covariables. The analyses did not show statistically significant differences between the groups when corrected for multiple comparisons.

#### Analyses of PTSD evaluations

The results of the CTQ, PCL-5, PHQ-9 evaluations and BAI were tested for normality of distribution using the Kolmogorov‐Smirnov or Shapiro‐Wilk tests. We used the Student's T test to assess the existence, or not, of statistically significant differences among the means of the total scores of each instrument, in both groups—except PCL-5 which is exclusive to the PTSD group. All statistical analyses of instrument scores were performed using SPSS software 20th version (SPSS, Chicago, IL, USA). The p-value < 0.05 was considered statistically significant.

### Ethical approval

The present study was approved by the Research Ethics Committee of the Pontifícia Universidade Católica do Rio Grande do Sul (number 57526716.1.0000.5336).

## Results

### Sample description

There were no significant differences between Control and PTSD groups' age, schooling, socioeconomic status and IQ. The BAI, PHQ-9 and CTQ scores were significantly higher for PTSD participants, relative to Controls (Table 1).

### Resting state fMRI results: negative connectivity associated with PTSD

**Figure 1:**
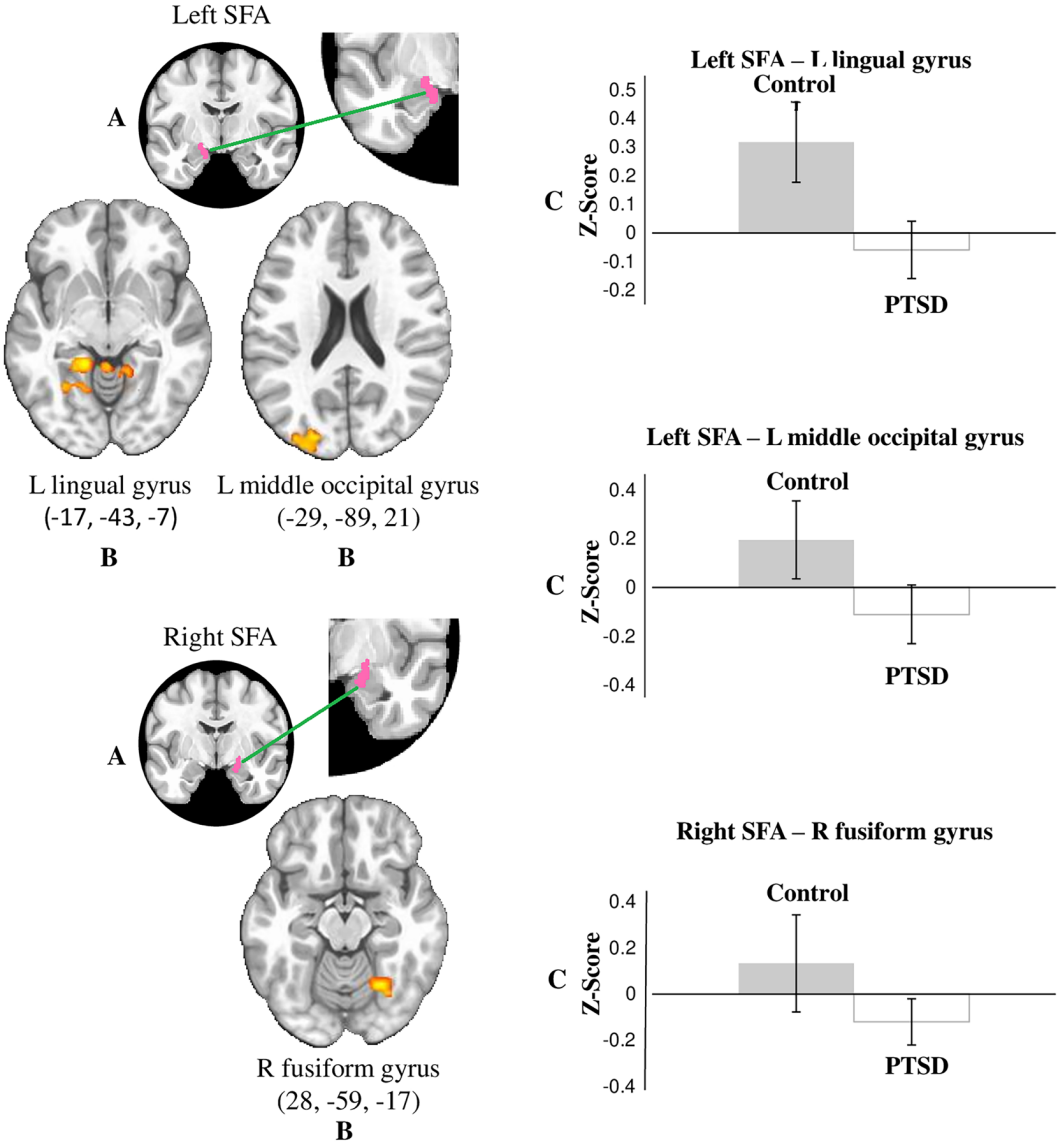
Areas of negative correlation between functional connectivity for left and right SFA and PCL-5 in the PTSD group. (**A**) Illustration of seeds, filled in pink (seed is magnified for better visualization); (**B**) overlays for the areas of the brain that showed significant association for the SFA, for PTSD PCL-5 scores (p < 0.05 corrected for multiple comparisons); (**C**) bar graphs show the values of the Z scores for connectivity between the regions shown in the overlays in (**B**). Standard error bars are shown for Z values.

The results showed PTSD was negatively associated with connectivity indices between the SFA and three posterior brain regions; the association was significantly different from controls. The pairs of regions that showed a significant difference were: (1) right SFA and right fusiform gyrus; (2) left SFA and left lingual gyrus; and (3) left SFA and left middle occipital gyrus (Fig. 1). The correlations among SFA and the three brain regions are reported in Table 2. No other statistically significant differences were found for the remaining amygdala seeds.

**Table 2:** Superficial amygdala (SFA): areas of negative association of SFA connectivity with PTSD group PCL-5 scores.

Seed	Cluster labels	Voxels (n)	Volume (μl)	BA	Peak MNI coordinates			Mean Fisher's *z*				
								PTSD group			Control group	
					x	y	z	Mean	SD		Mean	SD
Left SFA	LMOG	44	1886.5	19	− 29	− 89	21	− 0.1103*	0.1123		0.1955	0.1049
	LLG	116	4973.5	36	− 17	− 43	− 7	− 0.0588*	0.0845		0.3175	0.1595
Right SFA	RFG	44	1886.5	37	28	− 59	− 17	− 0.1199*	0.1064		0.1331	0.096

*BA* brodmann area, *MNI* Montreal Neurological Institute, *SD* standard deviation, *PTSD* posttraumatic stress disorder, *RFG* right fusiform gyrus, *LLG* left lingual gyrus, *LMOG* left middle occipital gyrus.

*Means statistically different from 0 in one sample t-test. All group differences represent significant differences between negative mean correlations (connectivity) in the PTSD group and the positive correlations in the control group. Cluster labels are for the regions that showed the significant difference in connectivity with the seeds. Differences are significant after correction for multiple comparisons (P < 0.05).

**Figure 2:**
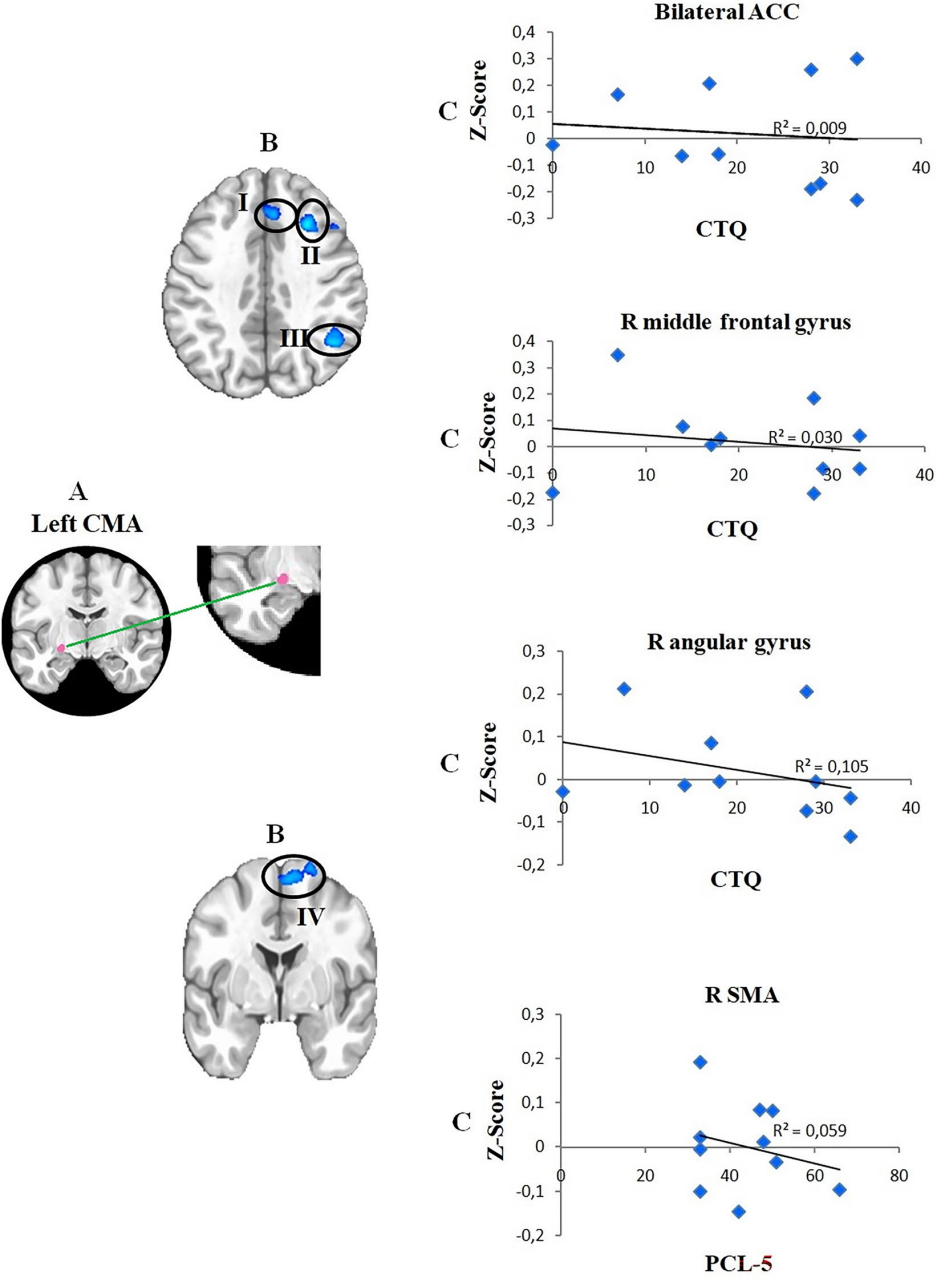
Areas of negative correlation between functional connectivity for left CMA and CTQ in the PTSD group. (**A**) Illustration of seeds, filled in pink (seed is magnified for better visualization); (**B**) overlays for the areas of the brain that showed significant association for the CMA, for PTSD CTQ scores (p < 0.05 corrected for multiple comparisons); (**C**) the scatter plots on the right show the relationship between the connectivity scores for the CMA and the associated regions. I = Bilateral anterior cingulate cortex (ACC); II = Right middle frontal gyrus. III = Right angular gyrus; IV = Right supplementary motor area (SMA); *R* right, *L* left, *CTQ* Childhood Trauma Questionnaire—total score, *PCL-5* Posttraumatic Checklist-5—total score.

For the PTSD group, the results show statistically significant negative associations between functional connectivity and PTSD symptomatology (PCL-5); there was also a negative correlation with childhood trauma (CTQ). In sum, results show that the higher the symptomatology and trauma scores, the lower the individual connectivity score. The total CTQ score was negatively associated with the connectivity score among the left CMA and a prefrontal cluster that included bilateral anterior cingulate cortex (ACC) and right middle frontal gyrus. The CTQ scores also showed a negative correlation with connectivity between left CMA and right angular gyrus. The PCL-5 scores showed a significant negative correlation with connectivity between the left CMA and the dorsal portion of the frontal lobe (supplementary motor area) (see Table 3 and Fig. 2).

**Table 3:** Centromedial amygdala (CMA): areas of negative association of CMA connectivity with PTSD group CTQ scores.

Cluster labels	BA	x	y	z	Cluster voxels	Volume (μl)
Bilateral ACC	32	4	37	25	79	3387.12
Right MFG	9	34	21	42	48	2058
Right AG	40	49	− 54	42	61	2615.37
Right SMA	6	10	− 6	67	72	3087

*BA* brodmann area, *ACC* anterior cingulate cortex, *MFG* middle frontal gyrus, *AG* angular gyrus, *SMA* supplementary motor area.

## Discussion

Our study showed a significant negative correlation between symptoms for PTSD caused by trauma in adulthood and brain connectivity between the SFA and three posterior brain regions: the right fusiform gyrus, the left lingual gyrus and the left middle occipital gyrus. Thus, the results suggest an overall pattern of increased PTSD symptomatology was associated with weaker functional connectivity between the amygdala and occipital, inferior parietal/temporoparietal and prefrontal regions. The direction of association between symptoms and strength of the functional connectivity corroborates the literature, which shows a pattern of results in the direction of worse symptoms, weaker amygdala functional connectivity in PTSD. More specifically, meta-analyses of brain imaging studies of rs-fMRI consistently show hypoconnectivity (or weaker connectivity) of the amygdala in association with PTSD^10,56,57^, including the regions described in the present study.

PTSD has been consistently associated with dysfunctions in a specific fronto-limbic network^58–62^ and with differences in brain function of occipital lobe regions^63–66^. For example, war veterans with PTSD showed reduced volume of gray matter in the left occipital lobe relative to veterans with no PTSD. The differences in volume correlated negatively with the severity of PTSD symptoms^63^. PTSD is consistently linked to altered basolateral and centromedial amygdala connectivity patterns^39–43^. A study of all three amygdala complexes in patients with dissociative PTSD identified BLA-Insula connectivity differences associated with clinical evaluations, but no differences associated with the SFA^38^. BLA and CMA complexes are closely linked to the learning of fear (BLA) and to the responses to this emotional learning (CMA)^33^.

The SFA complex is postulated to be involved in processing socially relevant information. As stated above, the connections of the SFA and olfactory cortex are well-known^18,37^. The interaction between SFA and olfaction has been associated with changes in emotional states^18^. Yet, there is evidence that it interacts with posterior regions associated with visual processes: A study of acute stress and rs-fMRI showed that SFA connectivity with the occipital lobe is stronger relative to the BLA and CMA connectivity with that same lobe^67^. Other studies have identified changes in bilateral SFA activity evoked by facial expressions: the SFA complex selectively captures the social value of the sensory information received^37^. A crucial role for the SFA in social interaction has been postulated: large-scale coactivation analyses suggest the SFA is connected to brain networks involved in reward prediction and affective processes^18^. Our findings showed alterations in PTSD for the SFA connectivity with the lingual gyrus and the fusiform gyrus. The lingual and fusiform structures are involved in processing high-level visual information, including face recognition^68^ and facial expressions^69–71^. This finding is in line with others that have shown alterations in amygdala-fusiform connectivity in association with early-life stress, institutionalization^25,26,72^ and PTSD^73^.

Traumatic events modify people's perceptions about themselves, and may lead to increased negative beliefs about oneself, and about life in general^74^. PTSD affects social cognition^75,76^ thus compromising the ability to predict what others feel, think or believe^75^. The perception of emotion-related expressions and regulation and learning of fear are among the key components affected in PTSD^75,76^; fear learning, in its turn, develops by an association between stimuli (such as olfactory or visual, for example) and aversive outcomes^77^. Inappropriate regulation of fear in PTSD can be associated with an exaggerated reaction to stimuli or mild stressor^78^; animal models suggest there is a sensitization of responses in PTSD that leads to readily learning new fears and exaggerated reactions in PTSD^79^.

Our results showed an association between functional connectivity of the CMA and ACC, in PTSD. The CMA complex projects to the autonomic and motor centers of the brain stem^33,34^ and is associated with generating fear responses^33^. It is closely linked with the ACC, for example, which in its turn is functionally connected to areas involved in affective processing^80,81^. The ACC is intimately involved in the assessment of emotion, in learning from and in relation to emotions and in emotional regulation. The ACC has been shown to release information to the amygdala and the prefrontal cortex^82^, reducing the activity of the amygdala when it is triggered by the resolution of emotional conflicts^83^. Others^84^ described weaker connectivity between the amygdala and dorsal ACC (Brodmann 32) in adult individuals with PTSD and a history of childhood abuse. We may postulate that history of trauma in development leads to alterations in amygdala-ACC connectivity, which in its turn may be linked to later increased susceptibility to PTSD.

We also found functional connectivity between the left CMA and the right angular and middle frontal gyri (MFG) to be negatively associated with CTQ scores. The angular gyrus is a brain region involved in higher-order processes of communication and executive function, such as integrating multimodal information, manipulating mental information, solving problems and redirecting attention^85^. It is located in the posterior parietal cortex, at the junction of visual, spatial, somatosensory and auditory processing flows. Sensorimotor attributes converge to the angular gyrus, which in its turn is associated with processing perceptual details^86^ and making semantic and conceptual associations^86,87^. Studies show right angular gyrus is associated with objective recall of specific details of episodic memory. Stronger connectivity with the medial temporal lobe was shown during recovery of information, when compared to the left angular gyrus^88^. Previous studies have explored regional spontaneous brain activity (called regional homogeneity, or ReHo) changes in PTSD patients who suffered severe traffic accidents^89^. Relative to controls, participants with PTSD showed weaker right angular gyrus ReHo, and a negative correlation of right angular gyrus with CAPS scores. It is argued that aberrant ReHo may be related to memory dysfunction and intrusive thoughts and memories^89^.

A growing body of literature shows the right MFG is associated with the suppression of memory and motivated forgetting^90–92^. Sullivan et al. (2019)^93^ found that right MFG activity is interrupted with exposure to trauma. Exposure to trauma may result in difficult voluntary suppression of negative images, and it may affect MFG and memory suppression. PTSD symptomatology scores (PCL-5) were also associated with lower connectivity between the left CMA and the supplementary motor area (SMA). The SMA plays a role in the regulatory network of emotions^94^. It is involved in the preparation of motor movement^95^ but also in the processing of affective stimuli related with motional imitation^94^. The SMA has a primordial function when preparing muscles for movement, with the objective of reflecting an event with an important emotional charge through affective facial and body gestures^94^. A significant decrease in connectivity between the amygdala and SMA was found in association with recovery of implicit memories^96^. Depressed function of the SMA may be related to an inability to fight or flight, a common symptom of PTSD^96,97^.

We did not find a significant association between anxiety (BAI evaluation) and functional connectivity of the amygdala with areas of the brain, for PTSD versus controls. The brain imaging literature suggests that anxiety disorders are associated with differences in amygdala-related functional connectivity that involve brain regions linked to executive function, such as the dorsomedial prefrontal cortex, cingulate gyrus and superior frontal gyrus^22–24^.

Our study has limitations, and the results should be interpreted with caution. First, the predominantly female population limits our ability to generalize; however, there is a vast literature of PTSD studies composed mostly of males, e.g., war veterans. In that sense, female predominance may be more of a novelty rather than a limitation. Second, our findings can be attributed, in part, to the limited sample size and image acquisition method. We know that the small sample size reduces the statistical power. As we described in the Methods section, our criteria limited our ability to find participants within a limited period. We emphasize that our exclusion criteria were conservative because our rationale was that only a more homogeneous sample would allow for insight into specific brain alterations associated with PTSD in adulthood. Furthermore, our results corroborate previous findings using conservative correction for multiple comparisons. Finally, there is the challenge of imaging amygdala function. It is well-known that the investigation of amygdala function and connectivity in humans is prone to brain imaging artifacts, especially due to the small volume of the structure^13^. The investigation of the function of even smaller subregions is more susceptible to such artifacts. Nonetheless, in the present study, we aimed to explore the functional connectivity of the amygdala subregions, despite the technical challenges that may present. Moreover, to clarify whether functional connectivity of amygdala subregions is separable using this study’s imaging and postprocessing protocols, we provide seed-based functional connectivity maps of each subregion from the control and PTSD groups separately (see Supplementary Material S1).

The results may corroborate the larger understanding, gleaned from brain imaging data, that amygdala-related connectivity alterations in PTSD and anxiety disorders are underpinned by aberrant brain states. The degree to which alternate brain networks show aberrant connectivity may provide valuable information about the associated psychological processes that are affected, e.g., self-regulation in anxiety disorders, and perception and sensation in PTSD. The brain networks that underpin executive functions are affected in PTSD depending on the type of trauma^11^. In general, despite the limitations, the results suggest that continuing to unveil the brain bases at rest of PTSD, and its association with an array of symptoms, etiology, and traumas may yet fulfill the promise of discovery science^98^, allowing for comparability across studies of clinical populations^99^ and may yet inform clinical practice and psychotherapy.

## Supplementary Information

Supplementary Information.

## Data Availability

Data will be available on the International Data Sharing Initiative (IND, http://fcon_1000.projects.nitrc.org/index.html), starting on August 2022.
