# Supplementary material for: An exploratory study of resting-state functional connectivity of amygdala subregions in posttraumatic stress disorder following trauma in adulthood
Source: Sci Rep. 2022 Jun 10;12:9558. doi: 10.1038/s41598-022-13395-8 (PMC9187646; doi:10.1038/s41598-022-13395-8)

**Supplementary Table: Seed based functional connectivity maps of each subregion from the PTSD and Control groups, separately**

| **PTSD GROUP*** | | | | | | | |
| --- | --- | --- | --- | --- | --- | --- | --- |
| **Seed** | **Functional connectivity** | **Voxels (n)** | **x** | **y** | **z** |  | **Figure**** |
| Right SFA | Right Amygdala | 81 | -24 | 7 | -6 | Positive | A |
| Left SFA | Left Hippocampus | 48 | 24 | 7 | -10 | Positive | B |
| Right BLA | Right Fusiform Gyrus | 59 | -31 | 4 | -27 | Positive | C |
| Left BLA | Left Amygdala | 79 | 24 | 4 | -20 | Positive | D |
| Right CMA | Left Superior Medial Gyrus | 124 | -3 | -34 | 32 | Negative | E |
|  | Right Inferior Frontal Gyrus  (pars orbitalis) | 112 | -38 | -24 | -10 | Negative | F |
|  | Right Amygdala | 92 | -21 | 0 | -17 | Positive | G |
|  | Left Inferior Frontal Gyrus  (pars orbitalis) | 61 | 38 | -24 | -3 | Negative | F |
| Left CMA | Right Middle Cingulate Cortex | 158 | -3 | -27 | 32 | Negative | H |
|  | Left Amygdala | 111 | 17 | 4 | -17 | Positive | I |
|  | Right Insula | 58 | -38 | -17 | -6 | Negative | J |
|  | Right Precuneus | 48 | -3 | 67 | 25 | Positive | H |
| **Control GROUP*** | | | | | | | |
| **Seed** | **Functional connectivity** | **Voxels (n)** | **x** | **y** | **z** |  | **Figure**** |
| Left BLA | Left Amygdala | 72 | 24 | 4 | -20 | Positive | K |
|  | Right Putamen | 53 | -24 | -3 | 18 | Negative | K |
| Left CMA | Left Amygdala | 54 | 17 | 4 | -17 | Positive | L |

*The maps were generated by removing the effects of BAI, PHQ9, and CTQ on the connectivity of both groups.

**Supplementary Figure: Seed-based functional connectivity maps for each amygdala subregion for the PTSD group.

Supplementary Figure: Seed-based functional connectivity maps for each amygdala subregion for the Control group.


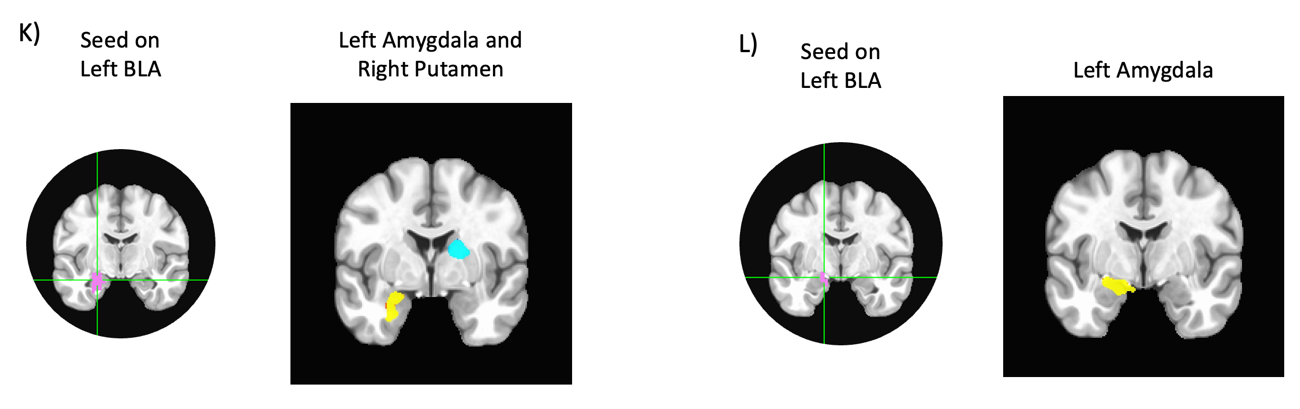

Supplement: Supplementary file 1 — Supplementary Information. [file 41598_2022_13395_MOESM1_ESM.docx]
